# Paper-Based Molecular-Imprinting Technology and Its Application

**DOI:** 10.3390/bios12080595

**Published:** 2022-08-03

**Authors:** Shufang Xu, Zhigang Xu, Zhimin Liu

**Affiliations:** Faculty of Science, Kunming University of Science and Technology, Kunming 650500, China; xsf2634098266@163.com

**Keywords:** paper-based analytical devices, paper-based molecular-imprinting technology, molecularly imprinted polymer

## Abstract

Paper-based analytical devices (PADs) are highly effective tools due to their low cost, portability, low reagent accumulation, and ease of use. Molecularly imprinted polymers (MIP) are also extensively used as biomimetic receptors and specific adsorption materials for capturing target analytes in various complex matrices due to their excellent recognition ability and structural stability. The integration of MIP and PADs (MIP-PADs) realizes the rapid, convenient, and low-cost application of molecular-imprinting analysis technology. This review introduces the characteristics of MIP-PAD technology and discusses its application in the fields of on-site environmental analysis, food-safety monitoring, point-of-care detection, biomarker detection, and exposure assessment. The problems and future development of MIP-PAD technology in practical application are also prospected.

## 1. Introduction

Paper-based analytical devices (PADs) are an emerging class of platforms that can operate without complex instrumentation, making them ideal for rapid analysis and deployment in areas lacking medical resources. Compared with traditional analytical techniques, paper-based devices have many advantages, such as low manufacturing cost, ease of operation, rapid detection, and ease of handling after testing. As a versatile substrate, paper has a porous structure that enables its surface to be easily modified and thus acquire a large surface-to-volume ratio. This makes it an excellent support for incorporating functional nanomaterials and a flexible platform for developing sophisticated analytical devices. Since 2007 when Whitesides’ group [1] patterned paper to create millimeter-scale channels, the applications of PADs have been reported in the fields of point-of-care testing [2,3], environmental monitoring [4,5,6], food-safety assessment [7,8,9], and biomedicine [10,11,12]. Currently, PADs are in paper chromatography [13,14], as lateral-flow test paper [15,16], as paper-based microfluidic devices [17,18], and in paper-spray ionization (PSI) [19,20], among others. However, despite the potential of PADs, their low accuracy and poor anti-interference ability under complex detection conditions limit their applications to a certain extent. Variations in the specificity of PADs in complex matrices such as blood and urine can lead to false-positive or false-negative test results, which reduce the accuracy and reliability of paper-based tests.

Molecularly imprinted polymers (MIPs) are a class of polymer-based biomimetic receptors. MIPs first combine template molecules and functional monomers with covalent or noncovalent bonds through self-assembly. Then they complete the polymerization in the presence of cross-linking agents and initiators. Finally, the template molecules are removed, thereby forming specific binding sites or cavities that are complementary in size and shape to the template molecule [21]. MIP exhibits good stability and resistance to matrix interference. Since 1931 when MIPs were first reported by Polyakov [22] using a silicon substrate, researchers have focused on the application of MIPs based on various analytical systems and sensors [23,24,25,26,27]. However, most current MIP analysis techniques require complex equipment and professional operators, which are difficult to operate during on-site inspections. They cannot be popularized and used in backward areas.

PAD detection systems, regardless of the sensing technology used, are particularly susceptible to various sources of interference in complex matrices. The addition of MIP into PADs can effectively reduce the nonspecific response of the detection system. In the multiple-signal readout mode of PADs, MIP as a biomimetic receptor can selectively bind to analytes in the sample and trigger changes in optical or electrochemical signals through recognition, thereby enabling quantitative measurements. In 2013, Yu’s research group [28] first investigated the potential of MIPs as an identification element in the assay of PADs. The group demonstrated their ability to enhance analytical performance, such as short response time, high selectivity and sensitivity, good reproducibility, and ability to perform high-throughput assays. Therefore, MIPs have received extensive attention as a specific recognition tool in the assay of PADs [28,29,30,31,32,33,34]. Furthermore, MIP-PADs enable the visual signal readout of results [35,36] without complex and expensive instrumentation. This review introduces MIP-PAD technology and its application progress in the fields of on-site environmental analysis, food-safety monitoring, point-of-care detection, biomarker detection, and exposure assessment. We also discuss the challenges in the practical application of MIP-PAD technology and development prospects.

## 2. Paper-Based Molecular-Imprinting Technology

Paper can be used as a substrate to immobilize MIPs. MIPs immobilized on paper can be used as recognition elements to capture target molecules in complex samples and design signal-sensing systems for these molecules [37,38], thereby improving the analytical performance of targets. Meanwhile, PADs are excellent substrates for the synthesis and modulation of MIP materials, facilitating the development of various flexible application modes while expanding their applications in portable devices. At present, the methods of preparing MIP-PADs primarily include in situ polymerization and post-introduction.

### 2.1. Preparation of MIP-PADs by In Situ Polymerization

**Figure 1:**
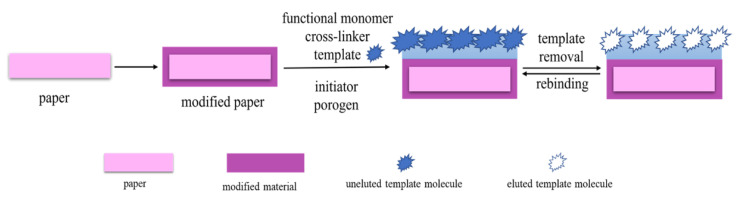
Preparation of MIP-PADs by in situ polymerization.

In situ polymerization can be used to directly synthesize MIPs on the surface of microfiber paper (Figure 1). This method involves the premodification of paper, the formation of template–monomer complexes, and in situ growth of MIPs. To associate paper with the MIP itself or other useful components during polymerization and application, the paper is pretreated with a silane coupling agent. The paper surface is rich in -OH groups, which can be easily modified using silane coupling agents to introduce various functional groups. Zhang et al. [39] selected Whatman filter paper as the carrier and modified the double bond with 3-(trimethoxysilyl) propyl methacrylate to promote the good bonding between the MIP layer and the paper surface.

Before MIPs are grown on paper, a template–monomer complex is formed by pre-assembly between the target molecule and the selected functional monomer. The polymerization reaction on the paper is then initiated in the presence of a cross-linking agent and an initiator under specific conditions, such as heat or UV light. Wang et al. [30] synthesized MIPs in microfluidic PADs (μPADs) under UV-light irradiation, enabling the simple, low-cost, rapid, and high-throughput detection of hazardous substances in real samples.

This method is most extensively used for the integration of MIP and PADs because of its simple operation and synergy between PADs and MIPs. However, MIP-PAD synthesis through this method involves immersing the paper in the solution for a long time during polymerization and elution. It also requires treatment such as shaking and sonication. These operations may reduce the stiffness and structural stability of the paper. Accordingly, the stability of MIP-PADs prepared by this method should be addressed in future studies.

### 2.2. Preparation of MIP-PADs by Post-Introduction Method

**Figure 2:**
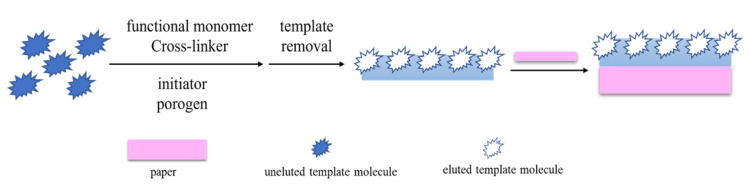
Preparation of MIP-PADs by the post-introduction method.

The post-introduction method is the direct synthesis of MIPs in a solution phase by traditional radical polymerization and sol–gel polymerization. Then the obtained MIPs are introduced onto PADs for subsequent applications (Figure 2). The polymerization-free strategy proposed by Díaz-Liñán et al. [29] provides a simple and convenient method of preparing MIP-PADs. This method avoids direct polymerization or elution on the paper and greatly reduces damage to the paper structure. Huang et al. [36] attach CdTe@SiO_2_@Si QDs-MIPs onto a hydrophobic polyvinylidene fluoride membrane by vacuum filtration to obtain a fluorescent test paper, which can be used to detect catechol in river water samples. Han et al. [40] synthesized MIPs with arginine as a pseudo-template on the surface of ZnFe_2_O_4_ particles and then added their complexes as a core substrate to the recognition region of μPADs. By combining molecular-imprinting technology and microfluidic paper chip, a new type of sensing platform is constructed using a smartphone, which realizes the portable detection of microcystins.

## 3. Paper-Based Molecular-Imprinting Technology

Devices based on paper-based molecular-imprinting technology are attracting considerable attention due to their rapid detection, low cost, and ease of operation. They enable rapid testing in a range of fields, such as on-site environmental analysis, food-safety monitoring, point-of-care detection, biomarker detection, and exposure assessment.

### 3.1. On-Site Environmental Analysis

Toxic and harmful pollutants in the environment are increasing, thereby inflicting certain harm to the survival of humans and animals. Thus, the demand for environmental-pollutant detection is increasing daily. However, for samples such as organic contaminants and pesticides in water and soil, the process of collection and transport back to the laboratory can cause fluctuations in the sample environment. Testing also remains largely limited to complex, centralized laboratories. Indeed, processing such samples is time-consuming and requires a large number of staff with technical expertise. Therefore, researchers are striving to explore and develop paper-based molecular imprinting techniques that are inexpensive and can detect environmental pollutants in real-time on-site.

**Figure 3:**
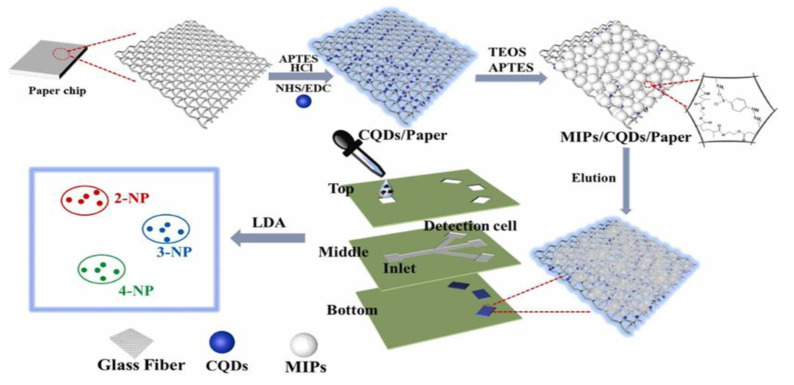
Schematic of the fabrication of a fluorescence sensor-array platform for the detection and discrimination of NP isomers. Reproduced with permission from Ref. [41]. Copyright 2022 Elsevier.

As important industrial raw materials, nitrophenols (NPs), are extensively used in various fields. Owing to the difficulties in NP degradability, they cause serious pollution to the environment and are classified as toxic pollutants and hazardous wastes in many countries. NPs have three isomers, namely 2-, 3-, and 4-NP. The 4-NP is much more toxic than the other two. Moreover, 2-NP and 4-NP are hydrolyzates of pesticides and are widely present in wastewater and food. They adversely affect the metabolism of an organism, although they are usually present in the environment as mixtures. Therefore, the identification of these three NP isomers in a simple and sensitive method is crucial to protecting the environment and human health. Zhu et al. [41] introduced a novel fluorescent sensor array μPAD for multi-analyte discrimination based on molecular-imprinting technology (Figure 3). Precise discrimination of the three NPs is achieved through MIP/carbon quantum dots (QDs)/paper sensor arrays. Furthermore, the discriminative ability of the platform is evaluated in a mixture of NP isomers, validating the utility of the device through the identification of dead-zone samples and achieving 100% accuracy. Qi et al. [42] proposed a molecular imprinting technique based on a spinning paper-based microfluidic chip to detect phenolic pollutants, as well as to perform the qualitative and quantitative analyses of 4-NP and 2,4,6-trinitrophenol. Under optimal conditions, the proposed sensor exhibits high sensitivity and selectivity. The limits of detection (LODs) are 0.097 and 0.071 mg/L respectively, enabling the rapid testing of environmental and biological samples.

Pentachlorophenol is an organic pollutant that enters the environment as a by-product of industrial processes, causing great environmental problems. Methods such as thin-layer chromatography [43] and gas chromatography–mass spectrometry (MS) [44] have been reported for pentachlorophenol determination. However, despite their good sensitivity and specificity, these methods are costly and time-consuming. Sun et al. [45] established an MIP sensor on a screen-printed paper electrode. The paper-based photoelectrochemical sensor has the characteristics of low cost, short absorption time, high precision, good stability, and reproducibility. It can be used to detect pentachlorophenol in real samples. Furthermore, Nie et al. [46] detected p-nitroaniline by using fluorescent molecularly imprinted paper strips based on nitrogen-rich QDs with an LOD of 1.65 nM, providing a simple, fast, and reliable assay for p-nitroaniline detection. Chi et al. [47] detected perfluorooctanesulfonic acid by using molecularly imprinted polyaniline on paper substrates with an LOD of 1.02 ng/L. The low cost, excellent sensitivity, and selectivity of this device indicate its potential for the large-scale monitoring of wastewater.

Neonicotinoids are the most extensively used insecticides in the world, causing paralysis and death of pest organisms. Thus, the real-time monitoring of neonicotinoid pesticide residues is highly significant to food security and the sustainable development of the ecological environment. In recent years, various analytical methods such as capillary electrophoresis, high-performance liquid chromatography, enzyme-linked immunosorbent assay, and electrochemical methods [48,49,50] have been used to detect neonicotinoid compounds. Although these methods can achieve high-sensitivity detection, their high cost, time-consuming nature, and complex sample preparation hinder their widespread application. Therefore, a simple, rapid, and inexpensive analytical technique to identify neonicotinoid compounds on-site needs to be developed. Zhao et al. [51] developed a paper-based surface-enhanced Raman scattering sensing platform based on a mixture of 3D silver dendrites and molecularly imprinted for the quantitative determination of neonicotinoids. The device exhibits ultra-high specificity and sensitivity for imidacloprid detection with an LOD as low as 0.02811 ng/mL.

In addition to testing for neonicotinoids, researchers have also tested other pesticide residues. Liu et al. [52] used a sensitive chemiluminescence detection method for the first time to detect dichlorvos on a paper device of MIPs. The paper-based chip has specificity and selectivity for dichlorvos detection in samples with an LOD of 0.8 ng/mL. Wang et al. [53] developed a simple paper-based molecularly imprinted photoelectrochemical sensor for S-fenvalerate detection with an LOD of 3.5 × 10^−9^ mol/L. Wang et al. [54] successfully developed a novel MIP-grafted paper-based multi-disk micro-disk plate and performed the high-throughput chemiluminescence detection of 2,4-dichlorophenoxyacetic acid. Vodova et al. [55] combined UV-induced fluorescence spectroscopy and MIP–PADs to detect selected commercial pesticides (ZATO 50 WG, TITUS WG 25) from pesticide-sprayed apples and tomatoes for the first time. These new MIP paper devices are particularly suitable for the field testing of pesticide residues such as pesticides in remote areas.

### 3.2. Food-Safety Monitoring

Food safety is closely related to human life and health and is drawing the attention of all countries in the world. In the food industry, the quality control of food products is very important before the product reaches the market, that is, from the production stage to the packaging stage. Food contaminated with viruses, bacteria, parasites, heavy-metal ions, pesticides, and other adulterated chemicals can exert serious human health and economic impacts. However, in the field of food safety, the matrix is complex, the content of heavy metals and the amounts of additives added are small, and the detection method needs to have good selectivity, low LOD, high sensitivity, and easy operation. These are the advantages of paper-based molecular-imprinting technology.

**Figure 4:**
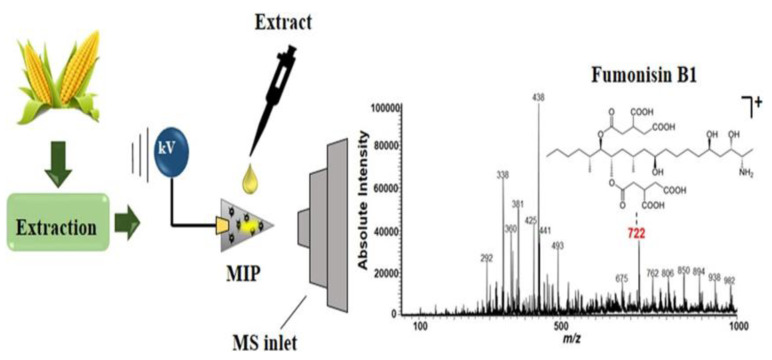
MIP-PSI-MS for the analysis of FB_1_ in maize samples. Reproduced with permission from Ref. [59]. Copyright 2022, Elsevier.

Maize is one of the most important crops worldwide due to its economic importance, nutritional value, and adaptability to different climates and regions. However, among cereals, corn is the food most susceptible to mold infection and has a relatively high content of fumonisin B_1_ (FB_1_). FB_1_ is a polyketide comprising 28 homologous compounds and is primarily produced by Fusarium spp. FB_1_ severely damages agriculture, food, and health due to its widespread presence and toxicity. The most commonly used detection methods for FB_1_ are chromatography-based methods and immunoassays, gas chromatography, and liquid chromatography coupled with MS [56,57,58]. However, these techniques require complex procedures, high solvent consumption, and expensive instrumentation. Accordingly, Ramalho et al. [59] proposed a new method of MIP combined with PSI-MS to improve the sensitivity of FB_1_ analysis in maize samples (Figure 4). The precision and accuracy of all analyses are below 5.5%. Finally, the MIP-PSI-MS method is used to quantify the FB_1_ content in commercial maize samples.

Additives are often added to food to improve its, color, aroma, and taste, as well as to meet the needs of antiseptic, fresh-keeping, and processing technology. However, excessive use may exert negative health effects, including the creation of gastric tumor precursors and DNA damage. It is commonly known that 17β-estradiol (17β-E_2_) is used to reduce production costs and increase profitability in the meat and dairy industries. However, the long-term exposure of 17β-E_2_ to the human body through the food chain can cause accumulation effects, as well as some endocrine and reproductive effects. With the improvement in people’s requirements for food safety, the residue of 17β-E_2_ is attracting increased research attention. The detection methods of 17β-E_2_ include instrumental analysis [60], among others. However, instrumental-analysis methods require expensive equipment and preliminary procedures for sample purification and are unsuitable for the rapid and real-time detection of 17β-E_2_. Xiao et al. [61] established a 17β-E_2_ detection method based on MIP-grafted paper. Their method has the advantages of simple and rapid operation, high sensitivity, and good selectivity and can be widely used to screen positive samples. Fan et al. [62] developed a three-dimensional graphite paper-imprinted electrochemical sensor for the selective recognition and sensitive detection of tert-butyl hydroquinone (antioxidant) with an LOD of 1.2 × 10^−8^ mol/L. The template molecule can be recognized from its analogs with high sensitivity. Chi et al. [63] prepared a nanoparticle for 3-chloropropane-1,2-diol (3-MCPD) detection by electrodepositing or electropolymerizing Prussian blue, platinum nanoparticles, and MIPs on the surface of electrochemically modified graphite paper. The sensor is also capable of detecting 3-MCPD in vegetable oil samples with simple, economical, rapid, and highly selective operation. Da Silva et al. [64] developed a sensitive electrochemical sensor based on molecularly imprinted polypyrrole film electropolymerized on graphite paper electrode (PE). The sensor exhibited high stability and good reproducibility and was successfully applied for the determination of LAC in whole and LAC-free milk samples.

Heavy-metal ion pollution has become an important global problem over the years due to its high risk to human health and the environment. Mercury is a highly toxic and harmful heavy-metal pollutant that threats the human body, such as movement disorders and coronary heart disease. Meanwhile, copper is an essential trace element for organisms and is closely related to human health. However, when the copper content in the body is too high, it imposes a burden on the liver and other organs, leading to metabolic disorders, liver cirrhosis, and other diseases. The quantitative analysis of copper and mercury ions in food monitoring is attracting considerable attention. Among them, the emerging paper-based microfluidic technology has unique advantages in the detection of heavy-metal ions. Qi et al. [65] developed a three-dimensional origami ion-imprinted μPAD for the multiplex detection of Cu^2+^ and Hg^2+^ by combining microfluidics and ion-imprinting technology. The LODs are 0.035 μg/L (Cu^2+^) and 0.056 μg/L (Hg^2+^) respectively. Wang et al. [66] proposed an ion-imprinted polymer-grafted paper-based fluorescent sensor based on QDs for Cu^2+^ detection.

### 3.3. Point-Of-Care Detection

Point-of-care testing refers to the rapid detection and analysis technology carried out at the bedside of patients. It is extensively used in hospitals in developed countries abroad. As a type of detection technology with great potential, it saves many sample-preprocessing steps, as well as numerous cumbersome processes, such as the detection, data processing, and transmission of large-scale instruments and equipment. The rational use of medical resources can directly and quickly obtain reliable results, ensure that medical staff in the emergency ward can obtain accurate test results of critically ill patients the first time, attain more rescue time, and be able to track the test results promptly. Point-of-care testing plays an important role in healthcare and can be applied to large populations, including rural areas with limited medical facilities. The introduction of paper-based devices and molecular imprinting technology into point-of-care testing has further expanded its application. Kumar et al. [67] reviewed the latest progress in the application of nanomaterial-modified conductive paper in point-of-care diagnosis.

Glycoproteins play crucial roles in various biological events, such as growth control, cell division, signal transduction, and cell migration. The occurrence of diseases is closely related to glycoprotein levels. Therefore, various glycoproteins have been identified as biomarkers in point-of-care testing. Since the COVID-19 pandemic caused by the severe acute respiratory syndrome coronavirus 2 (SARS-CoV-2) in 2019 brought dramatic changes to the world, many researchers have explored the detection of SARS-CoV-2 spike glycoprotein methods. Ratautaite et al. [68] described the application of a polypyrrole-based sensor for the determination of SARS-CoV-2-S spike glycoprotein. Given the presence of high levels of interfering substances and low glycoprotein concentrations in complex biological samples, the identification and detection of glycoproteins require high selectivity and excellent sensitivity. Sun et al. [69] proposed a strategy combining MIPs and hybridization chain reaction onto a microfluidic-paper-based assay device for the ultrasensitive detection of the target glycoprotein ovalbumin (OVA). The method can be used to detect OVA within a wide linear range from 1 pg/mL to 1000 ng/mL with a relatively low LOD of 0.87 pg/mL, indicating its potential applications in point-of-care and other related fields.

Point-of-care testing can be used for drug determination and is becoming increasingly important in laboratories to obtain information on drug-therapy suitability, drug interactions, and adverse effects. Some commercially available immunoassays have been used in clinical studies for drug determination, such as enzyme-linked immunosorbent assay (ELISA) [70], enzyme multiplex immunoassay [71], and fluorescence polarization immunoassay [72]. Although these assays are specific, they have the disadvantages of cross-reactivity with endogenous compounds, requiring specific skill and equipment, short shelf life and single-use corresponding kits, excessive washing steps, and high cost. Therefore, sensitive, selective, rapid, low-cost, and efficient methods of detecting drugs in body fluids for point-of-care detection are urgently needed. Akbulut et al. [73] reported an efficient, selective, rapid, and low-cost analytical method of detecting propranolol in human plasma samples by combining a molecularly imprinted Whatman paper with a UV-visible spectrophotometer.

**Figure 5:**
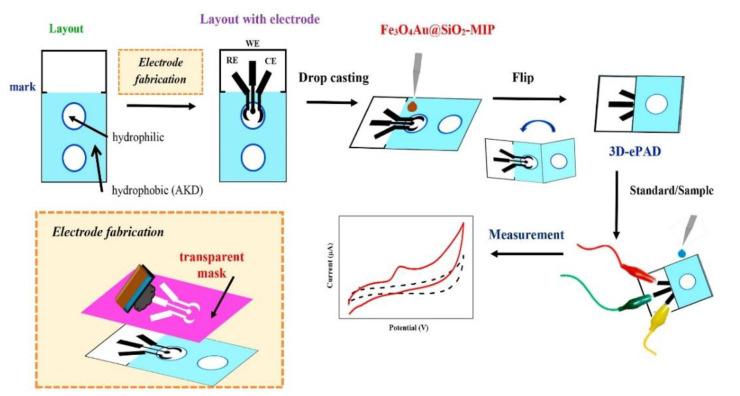
Preparation of Ser sensor and Ser detection using 3D-ePAD. Reproduced with permission from Ref. [74]. Copyright 2019, Elsevier. 3D-ePAD: Three-dimensional electrochemical paper-based analytical device.

Amatatongchai [74] designed an electrochemical paper-based device using a graphite screen-printed electrode modified with MIP-coated Fe_3_O_4_@Au@SiO_2_ for serotonin determination (Figure 5). The device is accurate, sensitive, and selective and is suitable for serotonin detection in samples such as urine. Chen et al. [75] also developed a paper-based electrical sensor with molecularly imprinted glucose-recognition sites for the determination of various glucose concentrations in bovine blood solutions. Pereira et al. [76] developed an MIP paper-based platform to detect β-amyloid, a biomarker of Alzheimer’s disease present in blood. These new MIP-PAD sensors have great potential for point-of-care detection applications.

### 3.4. Biomarker Detection

Biomarkers refer to specific biomolecules, such as DNA, proteins, enzymes, lipids, and carbohydrates that can reflect pathogenic factors or poisons from exposure to effect in the process. Biomarkers can be used for disease diagnosis and disease staging, as well as to evaluate the safety and efficacy of new drugs or treatments in target populations.

Biomarkers are simple to obtain and have wide-ranging sources, which are convenient for clinical application, especially in primary hospitals. Early detection of multiple biomarkers can improve the sensitivity and specificity of diagnosis. It has great value in judging disease severity, early intervention, guiding the application of antibiotics, and evaluating the efficacy of anti-infection agents. However, quantifying biomarkers is not easy because of their low concentration and the complexity of coexisting substances in biological fluids. Indeed, quantifying biomarkers in biological matrices face great challenges and thus requiring ultrasensitive detection. Accordingly, researchers have developed paper-based molecular-imprinting technology to detect biomarkers.

**Figure 6:**
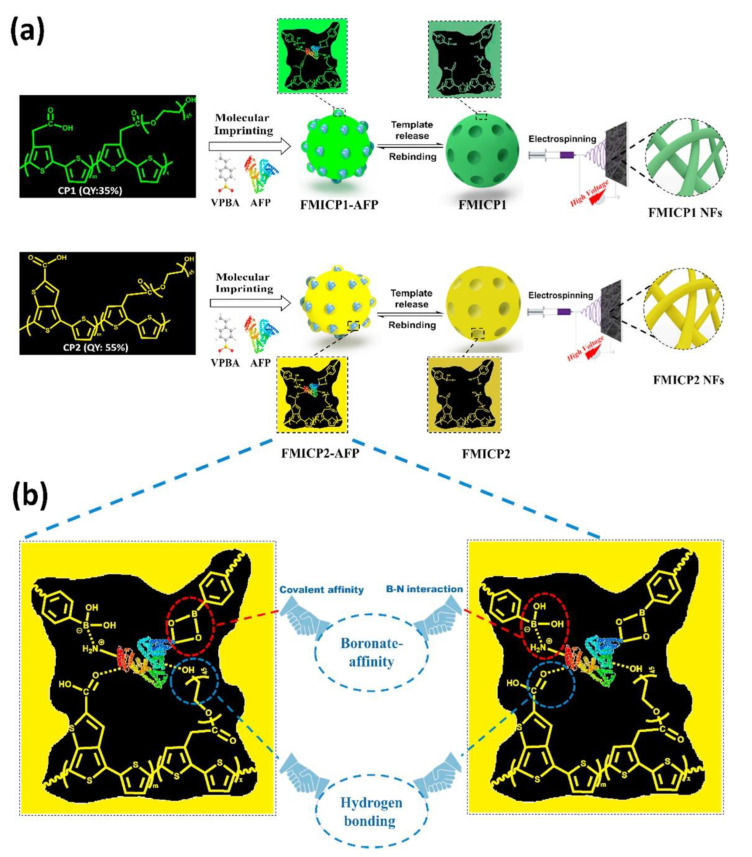
Principle and Strategy of FMICPs and FMICP NFs Biomarkers Assay: (**a**) Synthesis of the conjugated polythiophenes linked—molecular-imprinting strategy and fabrication of their fluorescent nanofibers using an easy and low-cost electrospinning approach, as well as their interactions with AFP biomarker. (**b**) Mechanism of dual-emission CPs linked with boronate-affinity molecular-imprinting strategy. Reproduced with permission from Ref. [77]. Copyright 2020, Elsevier. Notes: VPBA: 4-vinylphenylboronic acid, AFP: Alpha-fetoprotein.

The development of ultrasensitive, low-cost, and easy-to-use methods for the early detection of cancer biomarkers is of great interest. ELISAs are globally recognized clinical diagnostic assays because of their simple procedure, direct readout, and high feasibility. However, the high cost of antibodies, strict storage conditions, long reaction times, and cumbersome washing procedures limit the use of this method. Tawfik et al. [77] developed a widely applicable assay (Figure 6). Its analytical quality factor is comparable to that of ELISA while taking advantage of the many unique advantages of paper-based diagnostic platforms. The enzyme-initiated catalytic signal amplification step is replaced with signal amplification by conjugated polymers (CPs). CPs are converted into fluorescent molecularly imprinted conjugated polymers (FMICPs) through molecular self-assembly with an imprinting strategy. This fluorescent molecularly imprinted conjugated polymer is the first to produce highly fluorescent, flexible, and stable nanofibrous structures that can generate ultra-high, surface-area-to-volume ratios and numerous sensitive sites. Compared with traditional imprinted polymers, these polymers combine the strong specific binding sites of molecular imprinting with the unique characteristics of CPs to develop fluorescent molecularly imprinted conjugated polythiophene nanofibers (FMICP NFs) paper-based devices, which have an enzyme-free signal-amplification capability for biomarker detection.

Qi et al. [78] used a microfluidic paper-based electrochemical device on a MIP movable valve for the clinical detection of cancer biomarkers. This strategy can directly detect antigens by using MIP on paper-based devices, thereby greatly reducing the cost during clinical trials and the tedious washing process. It also eliminates the need to consider the preservation of antibodies in ELISA. This feature makes the chip suitable for on-site home processing or commercial products. The device is inexpensive, easy to prepare, and can provide reliable analysis compared with ELISA.

The determination of protein biomarkers plays an important role in the prediction of diseases, such as acute renal failure. Rypar et al. [79] coated a layer of polydopamine MIPs on distance detection-based microfluidic paper (DμPAD). Under alkaline conditions and in the presence of oxygen, polydopamine MIP-DμPAD is systematically investigated for the selective determination of chymotrypsinogen as a protein biomarker in urine within a linear concentration range of 2.4–29.2 μM (*R*^2^ = 0.9903). The corresponding relative standard deviations range from 2% to 11%, the LOD is 3.5 μM, and the LOQ is 11.8 μM. Li et al. [80] proposed a new strategy of fluorescent MIP test strips for ferritin detection, which can be used for the simple and visual detection of ferritin.

Biomarker levels in human fluids (including blood, urine, and saliva) are associated with various cancers, diseases, and degenerative diseases, such as Alzheimer’s disease, rheumatoid arthritis, Parkinson’s disease, and cardiovascular disease. Elevated levels of 3-nitrotyrosine (3-NT) in biological fluids are associated with Alzheimer’s disease, Parkinson’s disease, and pathologically related ischemia. The oxidation product of deoxyguanosine residues in DNA is 8-Hydroxy-2′-deoxyguanosine (8-OhdG). It is often used as a key biomarker of DNA damage. However, quantifying 8-OhdG in urine or serum is not easy because of its low concentration and the complexity of coexisting substances in biological fluids. The quantification of 3-NT in biological matrices is also difficult due to trace levels of endogenous 3-NT, which requires ultrasensitive detection. Nontawong et al. [81] reported a novel bimolecular imprinting electrochemical paper-based assay device for the simultaneous determination of 8-OhdG and 3-NT and assessed oxidation/nitrification biomarkers in urine and plasma samples. The LODs are 0.0138 μM and 0.0027 μM, respectively, with high selectivity and sensitivity.

Furthermore, Martins et al. [82] developed an MIP paper biosensor for 3-nitrotyrosine detection in human urine samples with an LOD of 22.3 nM. Tavares et al. [83] used PSI–tandem MS based on MIP substrates for the analysis of cocaine in oral fluid with an LOD of 0.27 ng/mL. Mendes et al. [84] proposed an analytical method of PSI-MS based on MIP-coated paper substrates for the detection and quantification of dopamine, sarcosine, and butyric acid in human urine without derivatization or complex sample pretreatment. The LODs are 0.24 μg/L, 0.5 pg/L, and 0.07 μg/L, respectively.

### 3.5. Exposure Assessment

In addition to applications in testing point-of-care, environmental contaminants, food safety, and biomarkers, paper-based molecularly imprinted devices can also be used for exposure assessment. Exposure assessment is the qualitative or quantitative evaluation of biological, chemical, and physical factors that may be exposed to humans or the environment through food intake or other relevant routes. Exposure assessments describe how a hazard enters the body, estimating the level of ingestion by different people. Exposure assessment regards biological, chemical, and physical factors in humans or the environment as recipients of environmental pollutants, making up for the deficiency in pollutant hazard assessment. Exposure assessment is a new way to control pollutants and provides new means for health-risk assessment.

**Figure 7:**
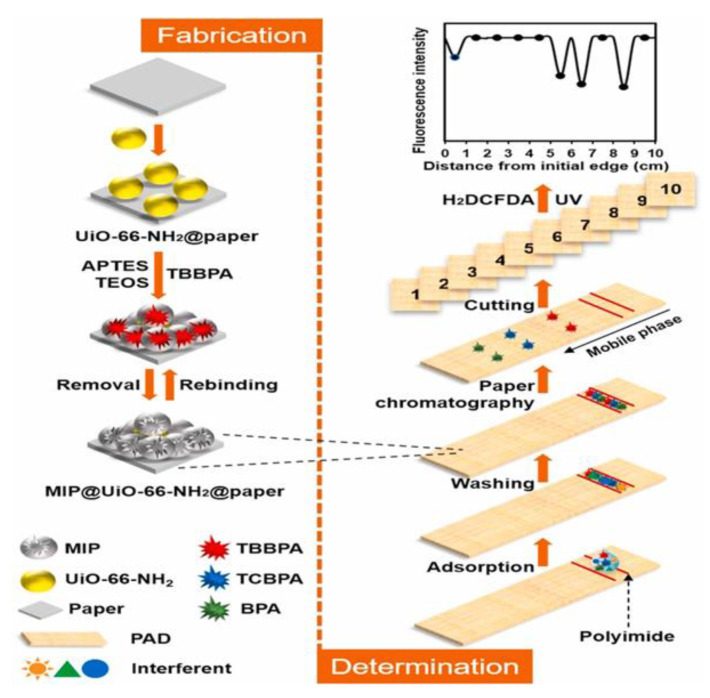
Schematic of the preparation of MIP@UiO-66-NH_2_@paper and associated analysis procedures. Reproduced with permission from Ref. [89]. Copyright 2021, Elsevier.

Bisphenol A (BPA) is extensively used to manufacture epoxy and polycarbonate plastics as a surface coating for cans, food containers, dental composites, and thermal paper. Its halogenated analogs, tetrabromobisphenol A and tetrachlorobisphenol A, are commonly used as reactants or additive flame retardants in various synthetic textiles, electronic devices, plastics, furniture, and building materials. Owing to the mass production and mass consumption of these consumer products, BPA and its halogenated analogs are ubiquitous. These compounds are also found in human serum and urine. BPA, tetrabromobisphenol A, and tetrachlorobisphenol A are persistent organic pollutants with endocrine-disrupting effects, cytotoxicity, genotoxicity, immunotoxicity, and neurotoxicity, as well as reproductive toxicity. Therefore, the simultaneous determination of bisphenols is critical to co-exposure assessment in humans. Conventional analytical methods for the simultaneous determination of these chemicals primarily rely on MS methods [85], but these methods have some limitations, such as complex instrumentation, high cost, and time-consuming preprocessing steps. Rapid methods for BPA determination have also been developed, including electrochemistry [86], fluorometry [87], and colorimetry [88]. However, they can be applied only to BPA and not for the simultaneous analysis of multiple compounds. Zeng et al. [89] used BPA as a template to prepare MIP based on metal–organic frameworks on filter paper (Figure 7). A MIP-based paper chromatographic separation and detection integrated μPAD is developed for the simultaneous determination of BPA and its halogenated analogs, and it is also used to determine the target chemicals in real dust samples.

Based on the molecularly imprinted curcumin nanoparticle platform, Mars et al. [90] developed a novel dual-modal μPAD for the electrochemical and fluorescence sensing of BPA. The sensing system has a wide linear range with LODs of 0.47 ± 0.2 and 0.62 ± 0.3 μg/L. The device has high selectivity to BPA, high stability during storage, and high reproducibility. Additionally, various real samples such as water, food, and plastic packaging are used to demonstrate sensor applicability. Kong et al. [91] combined the adsorption capacity of MIP film with ZnFe_2_O_4_ as a peroxidase mimetic and its colorimetric potential to prepare functional paper for BPA detection. This outstanding colorimetric sensor offers high selectivity, sensitivity, and stability. Visual quantitative inspection can also be achieved without any expensive instrumentation. Kamel et al. [92] first proposed a simple and ultra-low-cost disposable MIP paper potentiometric sensor for the determination of neutral BPA. The sensor displays a linear anion potential response within 0.5–13 μM, with an LOD of 0.15 μM, and exhibits good selectivity over other phenols.

Tobacco is extremely harmful to humans. Tobacco produces harmful gases and damages lung tissue. Long-term smoking is one of the most important causes of lung cancer. Tobacco contains a substantial amount of harmful substances such as nicotine and cocaine which can stimulate the blood vessels of humans and damage the arteries of the whole body. Accordingly, the assessment of tobacco-smoke exposure has high significance. Detection of the metabolites and chemical constituents of tobacco exposure, including nicotine, benzopyrene, and thiocyanate from body fluid specimens, such as urine, plasma, and saliva, is the target of smoking assessment. Among those biomarkers, cotinine, a major metabolite of nicotine, is widely recommended for the monitoring of tobacco smoke exposure because of its long half-life and distribution in various bodily fluids including blood, saliva, and urine. D’Aurelio et al. [93] introduced a sensor based on molecularly imprinted polymer nanoparticles and electrochemical impedance spectroscopy to detect trace amounts of cocaine with a detection limit of 0.24 ng/mL. The sensor enabled highly sensitive, portable, and cost-effective detection. For faster detection of harmful substances in tobacco, Larpant et al. [94] used noncovalent MIP to adsorb selectively cotinine on paper scaffolds. This study demonstrates that MIP paper stents can be used for the simple field sampling of cotinine and to assess tobacco-smoke exposure.

## 4. Conclusions and Perspective

MIP materials have the advantages of good selectivity, high sensitivity, and good stability. PADs are excellent tools for rapid analysis. The strategy of integrating MIP materials into PAD substrates can realize the miniaturization and integration of analytical devices. It has the outstanding advantages of low cost, easy portability, and simple operation, enabling rapid and high-throughput analysis.

MIP-PADs are emerging as paper-based integrated devices still in their early stages and with room for improvement. However, the technology is insufficiently mature, and users have low trust in it. Although MIP-PAD technology has rapidly developed over the past years, high sensitivity and specificity are still the primary indicators for MIP-PAD improvement. Conversely, MIP-PADs are usually synthesized using a single template molecule or ion, limiting their application in simultaneous multi-target analysis.

Paper-based molecular-imprinting technology will develop in the following four aspects in the future.

(1) The affinity for MIP is still lower than that of natural receptors, and many MIP materials show poor selectivity in aqueous media, which is unsuitable for the analysis of biological samples. In the future, the synthesis of MIP materials with higher affinity and hydrophilicity will also be developed.
(2) The preparation of multi-templated MIPs can generate distinct recognition cavities, allowing the simultaneous capture or removal of multiple analytes. Although challenging, the fabrication of multi-templated MIP-PADs holds promise for improving assay efficiency and multifunctional assays.
(3) The application research of paper-based molecular-imprinting technology in different fields is still worthy of attention. For the field of point-of-care and biomarker testing, developing simple, fast, and efficient method of processing and testing real-life samples is particularly important. In the field of food-safety testing, the functional integration of MIP and PADs should be improved, such as the development of a multiplexed analysis system that can identify multiple bacteria at one time, helping further reduce analysis time and cost. For the detection of environmental pollutants, most of the existing MIP-PADs are still in the laboratory stage of detecting synthetic samples due to their low selectivity, and MIP-PADs that can be applied in real environmental samples require further development.
(4) MIP-PADs can be combined with smartphones or through the development of various effective and portable intelligent signal-readout devices, which can be read anytime and anywhere, gradually become stable and generalized. They reduce the dependence on professional testing equipment and professional technicians. At the same time, realizing the development of MIP-PADs from pure qualitative analysis to semi-quantitative and fully quantitative with the help of smartphones will also become a trend.

Overall, future MIP-PADs will develop in the direction of simplicity, portability, intelligence, and practicality, providing an opportunity to promote global rapid detection and intelligent monitoring.

## Data Availability

Not applicable.
